# An Appeal for the Child

**Published:** 1906-07-21

**Authors:** 


### An Appeal for the Child.

For some years a small section of those who devote attention to matters of public interest have been impressed with the great importance of the child as a member of the community. "The boy is the father of the man" is an old and wise saying, yet, like many another saying which is full of teaching, it is often repeated, and perhaps admired for its wit, while the serious lesson it contains is allowed to pass unheeded. True though the saying is, the making of the man does not commence in boyhood. Deleterious influences, although acting at the very earliest periods of a child's existence, may cast their shadow over the remainder of its life. All efforts, therefore, which are directed towards the protection of our fellow human beings during the helpless stage of infancy should have our warm support.

One of the methods adopted to protect the infant is instruction of the mother. It is a fact beyond dispute that faulty feeding is, directly or indirectly, responsible for a large percentage of the mortality of infants during the first year of life. This faulty feeding is generally the result of ignorance. Recently we commented upon a letter giving wise and sympathetic advice to the mothers of Huddersfield, and since then a pamphlet following similar lines, but entering more fully into the subject of infant feeding, entitled "An Appeal for the Child," has appeared. The writer, Dr. John Grimshaw, says that "back to the land" is the cry of the social reformer, "back to the breast" of the guardian of infant life, and adds the cry of both is "back to nature." There are those, however, who are unable to provide the child with Nature's food, and a substitute has to be supplied. It is in the preparation of the substitute that the mother too often shows lamentable lack of knowledge, and that the need for widespread instruction displays itself. Although an advocate of milk depots, Dr. Grimshaw wisely places them in a secondary position. Yet he attaches importance to them, not only as the means by which milk free from causes of infection can be obtained, but on account of their educational value. An efficiently organised milk depot is not merely a milk dispensary where milk rendered free from danger can be obtained, but a school where ignorant and inexperienced mothers receive instruction upon infant feeding, since they not only obtain the milk, but are visited in their homes in order that the progress of the child may be noted. To provide every new-born infant that cannot be fed on the breast with pure, germ-free milk, and the mother with continued advice upon its use according to the varying and special needs of her child, is an ideal that may well be aimed at, yet, like so many other ideals, there are obstacles in the way of its attainment. Although only to the very needy is milk supplied free, such depots are not self-supporting.

That "the problem of infant mortality" depends upon "the trained intelligence, the inspired devotion, and the educated instincts of the mother" is a statement that contains a very large measure of truth; but the mother is by no means wholly responsible for the well-being of her infants. An ill-fed mother can scarcely have healthy children, and when born she cannot efficiently supply them with Nature's food. Responsibility rests with the father as well as with the mother. There is, no doubt, a considerable measure of real poverty where want in the home is the result of misfortune or ill-health on the part of the father, but if money that goes into the pockets of the bookmaker or the publican were bestowed upon the wife and the children we should hear much less of the need for meals in schools and still less of infant mortality.

